# Quadriceps Strength After Anterior Cruciate Ligament Reconstruction: Mechanisms of Weakness and Evidence-Based Rehabilitation Strategies

**DOI:** 10.7759/cureus.118798

**Published:** 2026-10-29

**Authors:** Hassan Zmerly, Rebecca Rauch, Erlis Velaj, Manuela Moscato, Rosaria Ipsaro Passione, Federica Dellafiore, Luigi Di Lorenzo

**Affiliations:** 1 Orthopedics, IRCCS (Scientific Institute for Hospitalisation and Care) Maria Cecilia Hospital and San Pier Damiano H, Faenza, ITA; 2 1st Unit of Orthopedics, Villa Erbosa, Bologna, ITA; 3 Faculty of Medicine and Surgery, Link Campus University, Rome, ITA; 4 Orthopedics and Traumatology, ASST (Azienda Socio Sanitaria Territoriale) Gaetano Pini Hospital, University of Milano, Milano, ITA; 5 Orthopedics, Villa Erbosa Hospital, Bologna, ITA; 6 Orthopedics, San Pier Damiano Hospital, GVM Care and Research, Faenza, ITA; 7 Life Sciences, Health, and Health Professions, Link Campus University, Rome, ITA

**Keywords:** anterior cruciate ligament reconstruction, arthrogenic muscle inhibition, muscle strength, quadriceps weakness, rehabilitation, return to sport

## Abstract

Impairment of quadriceps strength is a frequent and clinically important problem after anterior cruciate ligament (ACL) injury and reconstruction. This dysfunction reflects muscular, neural, surgical, and rehabilitation-related mechanisms and is not solely attributable to muscle atrophy. Despite restoration of joint stability, quadriceps weakness can persist and contribute to delayed functional recovery and reduced readiness to return to sport.

A comprehensive assessment should integrate clinical examination, objective isometric or isokinetic strength testing, rate of force development, functional performance, quality of movement, imaging investigations, and patient-reported and psychological measures.

Rehabilitation should be initiated preoperatively and continue postoperatively with a focus on control of effusion and restoration of extension, early quadriceps activation, progressive resistance exercise, appropriate open-chain loading followed by eccentric, plyometric, and sport-specific training. Neuromuscular electrical stimulation has supportive evidence as an adjunct, and blood-flow restriction training may be useful when higher loads may not yet be tolerated.

This narrative review summarizes current evidence on the mechanisms and rehabilitation of quadriceps weakness after ACL injury and ACL reconstruction, highlighting clinically relevant strategies for restoring strength and neuromuscular function.

## Introduction and background

Quadriceps weakness is a common and clinically important problem after anterior cruciate ligament (ACL) injury and ACL reconstruction (ACLR) and may persist well beyond the period typically considered sufficient for postoperative rehabilitation. Persistent quadriceps impairment is more complex than a simple loss of muscle mass [1-4].

Historically, this deficit was largely attributed to disuse atrophy. However, quadriceps weakness is now recognized as a multifactorial neuromuscular impairment resulting from the interaction of muscular, neural, surgical, and rehabilitation-related mechanisms [1]. Relevant contributors include muscle atrophy, intrinsic contractile dysfunction, arthrogenic muscle inhibition (AMI), altered spinal and corticospinal excitability, impaired voluntary activation, and surgical and rehabilitation-related factors [2-10].

These mechanisms are not independent but may interact and reinforce one another throughout recovery. For example, AMI and impaired voluntary activation can limit effective quadriceps recruitment, reducing force production and the mechanical stimulus required to maintain or restore muscle mass. Consequently, reduced muscle loading may contribute to further atrophy and weakness, potentially perpetuating a cycle of neuromuscular dysfunction. Similarly, alterations in spinal and corticospinal excitability may affect voluntary muscle activation, while intrinsic contractile dysfunction may further compromise force generation even when muscle activation improves [5-8,11-13].

Persistent quadriceps dysfunction is clinically relevant because strength recovery does not necessarily parallel the restoration of ligamentous stability or the passage of time after surgery. Evidence from systematic reviews indicates that deficits in quadriceps strength and voluntary activation may persist after ACLR, while morphological studies suggest that reduced muscle size accounts for only part of the observed impairment [2-4,9,10]. These findings support a broader pathophysiological model in which AMI, altered corticospinal excitability, intrinsic contractile dysfunction, graft-related morbidity, and insufficient progressive loading may interact and contribute to persistent weakness [5-8,11-13].

Furthermore, these deficits may persist despite successful restoration of mechanical joint stability and may adversely affect movement quality, functional recovery, and return to sport (RTS). Understanding the interactions among these mechanisms may help clinicians identify the predominant contributors to persistent quadriceps weakness and select more appropriate rehabilitation strategies.

Although individual mechanisms of quadriceps dysfunction have been investigated in previous systematic reviews and clinical studies [3,8,11-22], an integrated clinical synthesis remains valuable because these mechanisms frequently coexist and may influence the response to rehabilitation.

The aim of this narrative review is to critically synthesize current evidence on the mechanisms underlying persistent quadriceps weakness following ACLR, discuss their clinical implications for assessment, and examine evidence-based rehabilitation strategies aimed at restoring quadriceps strength, improving neuromuscular function, and facilitating a safe RTS.

## Review

Methodology

This narrative review was conducted using a structured literature search of PubMed/MEDLINE, Embase, Scopus, and Web of Science. The review covered literature published from January 2016 through September 2026, thereby capturing contemporary evidence on quadriceps dysfunction and rehabilitation after ACL injury and ACLR. The search strategy combined terms for the condition, impairment, assessment, and rehabilitation using Boolean operators: ("anterior cruciate ligament" OR "ACL") AND ("injury" OR "reconstruction" OR "ACLR") AND ("quadriceps weakness" OR "muscle strength" OR "muscle atrophy" OR "arthrogenic muscle inhibition" OR "voluntary activation" OR "neuromuscular dysfunction") AND ("assessment" OR "rehabilitation" OR "resistance training" OR "neuromuscular electrical stimulation" OR "blood flow restriction" OR "prehabilitation" OR "return to sport"). Search syntax was adapted to the requirements of each database.

Eligible publications were English-language, peer-reviewed human studies addressing ACL injuries or ACLR and at least one clinically relevant domain: mechanisms of quadriceps dysfunction, muscle morphology or neural activation, clinical assessment, strength recovery, therapeutic exercise, neuromuscular electrical stimulation (NMES), blood-flow restriction (BFR), prehabilitation, functional testing, or RTS. Randomized controlled trials, prospective and retrospective cohort studies, mechanistic studies, systematic reviews, meta-analyses, and clinical practice guidelines were considered. Case reports, non-English publications, studies without direct relevance to quadriceps function or rehabilitation after ACL injury/ACLR, and articles with insufficient clinical or mechanistic relevance were excluded.

Because the objective was an integrative narrative synthesis rather than a systematic review or meta-analysis, the search was used to identify the most relevant and methodologically informative literature across complementary study designs. No PRISMA flow diagram or quantitative pooling was planned, and a formal study-by-study risk-of-bias assessment was not performed. The final synthesis included 44 studies cited in the reference list that met the predefined eligibility criteria and directly informed the sections addressing underlying mechanisms, clinical assessment, rehabilitation strategies, and RTS.

The rationale for including multiple study designs was to connect biological mechanisms with clinically actionable rehabilitation evidence. Mechanistic and neurophysiological studies were used to explain persistent weakness; clinical trials and cohort studies were used to evaluate recovery and rehabilitation interventions; and systematic reviews, meta-analyses, and guidelines were used to contextualize the consistency and strength of the available evidence. Findings were synthesized thematically to link the underlying impairment to assessment and treatment decisions.

Pathophysiologic basis

Persistent quadriceps dysfunction is a multi-level neuromuscular impairment, not just postoperative muscle atrophy. Neural inhibition, structural muscle loss, impaired muscle quality, graft-related morbidity, pain, effusion, loading constraints, and behavioral factors may interact dynamically [4-11]. Important contributors include changes in muscle morphology and contractile function, arthrogenic muscle inhibition, neural and corticospinal changes, graft-harvest, and other surgical factors, and rehabilitation or behavior-related factors (Table 1) [12-18].

**Table 1:** Key factors for persistent quadriceps weakness post ACLR ACLR: anterior cruciate ligament reconstruction

No.	Contributing Factors
1	Muscle morphology and contractile dysfunction
2	Arthrogenic muscle inhibition (AMI)
3	Neural and corticospinal alterations
4	Graft harvest and surgical factors
5	Rehabilitation and behavioral factors

Muscle Morphology and Contractile Dysfunction

Muscle atrophy contributes significantly to quadriceps weakness, particularly during the early postoperative months, but it does not explain the entire deficit. Thomas et al. demonstrated a strong relationship between quadriceps cross-sectional area and strength at six months after ACLR [9]. Nevertheless, a systematic review by Birchmeier et al. found that, although quadriceps cross-sectional area or volume was generally smaller in the reconstructed limb, morphological loss explained only part of persistent weakness [10].

At the cellular level, postoperative muscle may also be qualitatively impaired. Tourville et al. reported skeletal-muscle contractile dysfunction at approximately six months after ACLR, indicating that force-generating capacity may remain abnormal beyond what would be predicted by muscle size alone [11]. These findings support an important distinction between muscle quantity and muscle quality [11]; patients with similar quadriceps cross-sectional area may demonstrate different force-generating capacity because of differences in neural activation and intrinsic contractile function. This distinction has therapeutic implications. Increasing muscle size without restoring maximal force, rapid force production, and task-specific recruitment should not be considered complete rehabilitation [14].

Arthrogenic Muscle Inhibition

AMI is a reflexive inability to fully activate musculature surrounding an injured joint despite an anatomically intact motor pathway. ACL rupture and reconstruction disturb the sensory environment of the knee through inflammation, effusion, nociception, and altered mechanoreceptor input. The resulting aberrant afferent signal changes spinal-reflexive processing and motor-neurone excitability, thereby reducing effective quadriceps neural drive. AMI can affect not only maximal force but also the ability to generate force rapidly and coordinate the quadriceps appropriately during functional loading [5,6].

Joint effusion is particularly relevant because relatively small changes in the intra-articular environment can modify afferent discharge without requiring major structural muscle damage. Clinically, the combination of effusion, pain, extension loss and an extensor lag should therefore be interpreted as an active neuromuscular problem rather than simply an expected inconvenience of surgery [5].

AMI can create a cycle of persistent weakness. Reduced muscle activation decreases mechanical loading, thereby reducing the stimulus for muscle growth and promoting atrophy. Weaker muscles then produce less force during rehabilitation and daily activities, further slowing recovery. This may explain why quadriceps strength does not always return spontaneously after swelling resolves [4,9,10].

Neural and Cortical Changes

Neural and cortical factors seem to be involved in the recovery after ACLR [7]. Neuroimaging and neurophysiological studies have reported changes in corticospinal organization and excitability after ACL injury and reconstruction [8]. In a study by Lepley et al., diffusion tensor imaging and transcranial magnetic stimulation (TMS) revealed differences in corticospinal-tract structure and excitability that could potentially influence motor recovery after ACLR [7]. De Brito Santana performed a meta-analysis of 14 TMS studies (n=381) and found higher motor thresholds in ACL-injured or reconstructed participants compared to healthy controls, with changes also reported in the contralateral limb [8].

These observations are relevant for the interpretation of the limb symmetry index (LSI). If unilateral ACL injury is coupled with bilateral neural adaptation or systemic detraining, the contralateral limb may no longer be a stable healthy reference.

Voluntary activation provides a clinically relevant link between central neural changes and force production. Its accurate quantification can therefore help characterize the extent of quadriceps dysfunction [15].

Surgical and Graft-Related Factors

The pattern of postoperative strength deficits varies with graft harvest. With quadriceps tendon (QT) and bone-patellar tendon-bone (BPTB) grafts, there is direct involvement of the extensor mechanism, whereas deficits are more specific to the flexors with hamstring tendon (HT) grafts. Holmgren et al. evaluated a matched cohort of 312 patients at 7 months and showed quadriceps LSI values of 67.5% to 75.1% after QT grafting, 74.4% to 81.5% after BPTB grafting and 84.0% to 89.0% after HT grafting [12].

Such differences should not be interpreted as the global inferiority of one graft. White et al. recently reported broadly similar graft failure, laxity and patient-reported outcomes for QT, HT and BPTB autografts in a meta-analysis of randomized trials [13].

Additional factors that may further reduce the achievable loading dose include concomitant meniscal repair, cartilage procedures, anterior knee pain, postoperative stiffness, and restrictions on weight-bearing or knee flexion. Thus, a perceived deficit in rehabilitation may sometimes be a reflection of an insufficient mechanical stimulus imposed by the necessary surgical precautions rather than solely reflecting inadequate muscle adaptation.

Rehabilitation-Related and Behavioral Factors

Persistent weakness may also be maintained by rehabilitation-related under-loading. Although initial protection is necessary, prolonged use of low-load closed-chain exercises without progressive knee-extensor overload may be insufficient to restore the strength required for running, jumping, and pivoting. Current guideline evidence supports combining open- and closed-kinetic-chain strengthening and including both eccentric and concentric work, rather than strictly avoiding open-chain knee extension [1,17].

Under-loading may also occur due to pain avoidance, fear of re-injury, low confidence, and poor adherence. Sell et al. conducted a systematic review and found that Anterior Cruciate Ligament-Return to Sport after Injury (ACL-RSI) scores changed over time after ACLR, indicating the changing nature of psychological readiness [19]. Psychological factors may affect exercise intensity, willingness to engage in high-force tasks with the knee, and RTS behavior [18].

Clinical assessment

Persistent quadriceps weakness after ACLR requires a comprehensive assessment that integrates strength, voluntary activation, and arthrogenic muscle inhibition assessment, functional performance, movement quality, and patient-reported outcomes [23-30]. Knee-extension strength can be measured with isometric or isokinetic dynamometry, whereas the rate of force development (RFD) provides information about the rapid force-generating capacity. Magnetic resonance imaging (MRI) and ultrasound analyses have been proposed to provide a comprehensive assessment of the thigh musculature [20,21].

Hop, jump, squat, and balance tests provide complementary functional information, although compensatory strategies may mask persisting weakness. Patient-reported measures should also be incorporated. The International Knee Documentation Committee (IKDC) Subjective Knee Form is a validated knee-specific measure of symptoms, function, and sports activity [31], while the Knee Injury and Osteoarthritis Outcome Score (KOOS) assesses pain, symptoms, activities of daily living, sports/recreation, and knee-related quality of life [32]. Psychological readiness can be assessed with the ACL-RSI scale, which was specifically developed to capture emotions, confidence, and risk appraisal related to RTS [33]. Strength should be compared with the contralateral limb and, where available, appropriate normative or absolute reference values, because bilateral weakness may result in falsely elevated symmetry (Table 2) [16,30].

**Table 2:** Main components of clinical assessment of quadriceps weakness after ACLR ACLR: anterior cruciate ligament reconstruction

No.	Assessment Methods
1	Clinical examination and Limb Symmetry Index (LSI)
2	Isometric and isokinetic strength testing
3	Voluntary activation and arthrogenic muscle inhibition (AMI) assessment
4	Rate of force development (RFD)
5	Functional testing and movement quality
6	Patient-reported outcomes and psychological assessment
7	Ultrasound and magnetic resonance imaging (MRI)

Prevention

Prehabilitation

When surgery is not urgent, it is better to perform ACLR with minimal effusion, full or near-full extension, good quadriceps activation, and greater strength than to start surgery with a profoundly inhibited limb.

Giesche et al. performed a systematic review on prehabilitation before ACLR and concluded that preoperative programs that include quadriceps strengthening, neuromuscular training, and restoration of range of motion may enhance preoperative function and might have a postoperative benefit; however, the optimal duration and content of these programs remains unknown [18]. The literature programs included generally ranged from 4 to 16 weeks; therefore, prehabilitation is better viewed as an individualized strategy than as a mandatory fixed-duration protocol [18].

Control of the Postoperative Joint Environment

Early priorities include restoration of extension, progressive flexion, reduction of clinically important effusion and pain, and recovery of active straight-leg raise without lag. Cryotherapy may help manage symptoms in the short term, especially to reduce pain, which may allow exercise [1,5].

Early Quadriceps Recruitment

Quadriceps isometrics should be initiated as tolerated, with terminal extension control, and progressive weight-bearing initiated as dictated by the surgical procedure. Patients with significant failure of activation, extensor lag, or inability to produce a meaningful voluntary contraction should be considered for early neuromuscular electrical stimulation (NMES) rather than an increase in the number of ineffective low-intensity voluntary contractions [22,23].

Treatment

The goal of treatment is primarily to develop a knee that can tolerate progressive training. Treatment of persistent effusion, marked anterior knee pain, flexion contracture, or extension lag that limits voluntary force generation should be combined with strengthening. Prolonged complete bed rest is generally not beneficial when surgical restrictions permit mobility, and the stimulation for rehabilitation should advance with adequate control of pain, effusion, and range of motion [1,5].

Progressive Resistance and Open Kinetic Chain Knee Extension

Restoration of quadriceps strength requires progressive increases in mechanical tension. Both closed-chain exercises (e.g., squats, split squats, step variations, and leg presses) and open-chain knee-extension exercises are important, because closed-chain tasks may allow significant contributions from the hip extensors and, in bilateral exercises, the contralateral limb.

Evidence from systematic review and clinical guidelines suggests that appropriately prescribed open- and closed-chain exercises can improve quadriceps strength without a clear clinically important increase in graft laxity [1,17].

When pain, effusion, graft type and surgical constraints allow, open-chain loading should be introduced in a protected range with progressive increases in range of motion, external load and contraction velocity. Dose modification should be guided by anterior knee pain, especially post BPTB or QT harvest [17].

Eccentric Training

Eccentric exercise is characterized by high force production at a relatively modest metabolic cost and can provide a powerful hypertrophic and strength stimulus. Milandri reported improvements in muscle and functional outcomes in a randomized controlled trial comparing eccentric versus concentric cycling during ACLR rehabilitation [24]. However, current evidence does not establish a single superior eccentric protocol.

Neuromuscular Electrical Stimulation

NMES is among the better-supported adjuncts for marked early quadriceps activation deficits. In a systematic review and meta-analysis of RCTs, Li et al. found greater quadriceps-strength recovery when NMES was added to standard rehabilitation, with larger effects when treatment was initiated early [22].

The intensity of NMES should be increased until it reaches the maximum comfortable level that causes a strong visible contraction. Device parameters differ across trials; therefore, achieving an adequate contraction is clinically more important than rigid adherence to a single waveform or parameter set [22,23].

Training With BFR

BFR training may provide a meaningful muscular stimulus at relatively low external loads and thus could be useful when heavier resistance is limited by postoperative pain, joint irritation, or tissue-healing constraints. The evidence remains inconclusive, with some studies reporting improvements in quadriceps strength or functional outcomes and others reporting limited or inconsistent benefits [25-27].

It is better to consider BFR as an adjunct or a bridge to traditional progressive resistance training and not as a replacement. Patients should be screened for relevant vascular or thrombotic risk, uncontrolled cardiovascular disease, and other contraindications.

Strength, Power, and RFD Rehabilitation

The patient may have reached peak torque, but still have a deficit in RFD. Therefore, the late stage of rehabilitation should shift from the goal of increasing strength to generating force rapidly and in sport-specific environments. Maximal strength is based on high-load training; afterward, RFD and power can be progressively improved by concentric exercises with the intention of speed, jumps (jumps and hops), and running accelerations/decelerations [14].

Plyometric training can progress from submaximal bilateral landing and pogo-type activities to unilateral jumping, repeated stretch-shortening-cycle activities, horizontal and multiplanar activities, deceleration, and finally, reactive change of direction. Progression should be based on the patient's ability to tolerate load without a clinically meaningful increase in pain or effusion, while maintaining adequate knee-extensor contribution rather than a return to a stiff-knee or hip-dominant strategy [1].

The first treatment objective is to create a knee that can tolerate progressive training. Persistent effusion, substantial anterior knee pain, flexion contracture, or extension lag may limit voluntary force output and should be addressed concurrently with strengthening. Once surgical restrictions permit movement, prolonged complete rest is generally counterproductive; the rehabilitation stimulus should progress while pain, effusion, and range of motion remain acceptably controlled [1,5].

Psychological and Return-to-Sport Integration

RTS should be considered the final phase of a progressive rehabilitation continuum rather than a clearance event based solely on time from surgery. Quadriceps recovery should be evaluated alongside hop and jump capacity, RFD, power, running and deceleration mechanics, sport-specific exposure, and psychological readiness [16,28,29].

In the Delaware-Oslo ACL cohort, Grindem et al. reported a higher reinjury rate among athletes returning to high-demand level-I sport and a lower reinjury rate with delayed RTS; within that cohort, each month of delay up to nine months was associated with a lower reinjury rate [28]. These observational findings support caution regarding early RTS but should not be interpreted as evidence that reaching nine months alone makes an athlete safe. Systematic evidence also indicates that conventional RTS test batteries have limited ability to predict a second ACL injury [29,30].

For athletes returning to pivoting sports, an LSI of at least 90% for quadriceps strength and hop performance is commonly used as a minimum benchmark, but it should not be interpreted as a stand-alone clearance criterion [30].

Physical capacity and psychological readiness should be assessed together during RTS decision-making. Fear of reinjury and low confidence may limit loading and participation, whereas high confidence does not necessarily indicate adequate physical recovery. Psychological readiness should therefore complement, rather than replace, objective measures of strength, power, movement quality, and sport-specific capacity [29].

Discussion

Persistent quadriceps weakness after ACLR reflects the interaction of muscular, neural, surgical, and rehabilitation-related factors. Restoration of mechanical knee stability does not necessarily indicate recovery of muscle function. Muscle atrophy contributes to strength loss, but impaired voluntary activation and intrinsic contractile dysfunction may also limit force production [4,9-11]. These findings support a rehabilitation approach that addresses both the barriers to quadriceps recruitment and the progressive loading required to restore muscular capacity.

The association between AMI and muscular atrophy is particularly relevant for clinical practice. Pain, effusion and altered joint afferent input may reduce quadriceps activation, limiting the mechanical stimulus achieved during exercise [5,6]. Thus, it is possible for a patient to regularly attend rehabilitation but not get enough effective loading to recover strength. Therefore, if weakness remains, the joint environment, voluntary activation, exercise intensity and adherence should be re-evaluated. Symptom management and strengthening should be combined, with exercise modifications based on the patient’s response and surgical restrictions.

Assessment also needs to be interpreted with care. The LSI is a practical measure of between-limb differences, but it may overestimate recovery when the contralateral limb is affected by detraining or bilateral neuromuscular changes [16,30]. Thus, apparently satisfactory symmetry may coexist with inadequate strength in the two limbs. Where appropriate reference data are available, strength testing should include absolute values, normalisation for body mass and comparison with relevant healthy populations. Functional tests provide additional information, but satisfactory hop performance may mask compensatory movement strategies. A combination of dynamometry, movement assessment, patient-reported outcomes and psychological measures can provide a more complete picture of recovery than any one test alone.

Maximal strength and rapid force production should also be distinguished. Recovery of peak torque does not necessarily indicate that the quadriceps can generate force sufficiently quickly during demanding activities [14]. This distinction helps explain why conventional strengthening alone may leave residual limitations during running, landing, and changes of direction. Later rehabilitation should progressively incorporate power, plyometric, deceleration, and sport-specific tasks while maintaining adequate knee-extensor loading and monitoring pain, effusion, and movement quality.

Graft-related differences further support individualized rehabilitation. Quadriceps tendon and BPTB harvesting directly involve the extensor mechanism and may be associated with greater early quadriceps deficits than hamstring tendon harvesting [12]. However, these differences should not be interpreted as evidence of overall graft inferiority, given the broadly comparable clinical outcomes reported in randomized-trial evidence [13]. Graft choice, donor-site symptoms, concomitant procedures, and postoperative restrictions should inform exercise progression. Patients with slower strength recovery may require a longer period of targeted loading rather than progression based solely on elapsed postoperative time.

Ongoing resistance exercise is an essential component of therapy. Open- and closed-chain exercises can be complementary in achieving quadriceps loading and their prescription should be guided by symptoms, healing constraints and functional goals [1,17]. NMES may be beneficial in the early rehabilitation phase where voluntary activation is very poor, but should be used to facilitate progression toward effective voluntary exercise [22,23]. In case of poor tolerance to heavier loads, BFR might provide an alternative. However, inconsistent findings prevent definitive conclusions on its benefits and optimal application [25-27]. Therefore, adjunctive treatments should be selected based on the patient's limiting impairment and evaluated by objective improvements in strength and function.

These considerations also apply to RTS decision-making. While quadriceps strength is a significant component of readiness, neither a symmetry threshold nor a fixed postoperative interval can determine safety of return alone. Observational data have suggested caution in returning to demanding sport too early, and systematic reviews have highlighted limitations in the predictive ability of conventional RTS test batteries [28,29]. Physical recovery should be coupled with psychological readiness and progressive exposure to sport-specific demands. Fear of reinjury may limit loading and participation, and confidence does not guarantee adequate physical capacity.

Taken together, the evidence suggests that persistent quadriceps weakness should be treated as a changing impairment profile rather than as a single deficit. Early after ACLR, effusion, pain, AMI, and failure of voluntary activation may be the dominant constraints; later, insufficient hypertrophy, graft-harvest morbidity, reduced maximal force, impaired RFD, and incomplete exposure to high-force or high-velocity tasks may become more important [4-14]. This has practical implications for rehabilitation: the intervention should be matched to the limiting mechanism and repeatedly re-evaluated with objective testing. NMES is most defensible when activation failure limits voluntary loading [22,23], BFR may serve as a temporary strategy when conventional high-load resistance is poorly tolerated [25-27], and progressive resistance, eccentric, power, plyometric, and sport-specific training become increasingly important as joint irritability resolves [1,14,17,24]. No single modality should therefore be viewed as sufficient across all phases of recovery.

The early postoperative phase illustrates the need to balance suppression of arthrogenic inhibition with avoidance of therapeutic under-loading. Criterion-based staging may be more informative than time alone: during approximately the first 0-6 weeks, restoration of extension, control of effusion and pain, normalization of gait, and recovery of voluntary quadriceps activation remain central, while a quadriceps LSI of approximately 60% has been proposed as an early milestone rather than a universal clearance threshold [34]. This framework is clinically useful because persistent inhibition can reduce the effective mechanical stimulus even when a patient appears adherent to rehabilitation. Accordingly, symptom control should facilitate progressive loading rather than delay it unnecessarily [35,36].

Adjunctive strategies should be interpreted according to the impairment they are intended to overcome. NMES is most relevant when voluntary activation is insufficient to generate an effective contraction; recent evidence also suggests effects on force steadiness, motor-unit behavior, and muscle-quality measures, although these findings remain based on relatively small cohorts [37]. Contralateral NMES may attenuate strength loss when loading of the reconstructed limb is restricted [38], and cross-education provides a complementary neural strategy: an RCT reported 28% and 31% greater quadriceps strength gains with concentric and eccentric training of the uninvolved limb, respectively, compared with control rehabilitation [39]. These approaches should therefore be considered temporary facilitators of recovery, not substitutes for progressive direct loading of the reconstructed limb. Early loading options may also include carefully selected co-contraction strategies that can generate meaningful quadriceps activation while limiting anterior tibial shear [40].

The evidence for BFR further emphasizes the importance of matching an intervention to the phase of recovery rather than assuming a uniform treatment effect. One meta-analysis of eight RCTs reported improvements in isokinetic strength (SMD 0.77), IKDC score (mean difference 10.97 points), and pain (SMD 1.52), without a significant improvement in quadriceps muscle volume [41]. In contrast, a more recent meta-analysis found no significant benefit for quadriceps cross-sectional area or MVIC during the first three postoperative weeks, while IKDC outcomes improved more consistently at 8-14 weeks [42]. A systematic review likewise highlighted heterogeneity across BFR protocols and outcomes [43], whereas a multicenter matched cohort reported superior early functional and IKDC outcomes through 12 weeks [44]. Taken together, these findings support BFR as a potentially useful bridge when high-load resistance is poorly tolerated, but not as a replacement for conventional progressive resistance training once adequate mechanical loading becomes feasible. This distinction is important for return-to-sport preparation, where restoration of maximal strength must ultimately be integrated with RFD, power, movement quality, sport-specific exposure, and psychological readiness rather than judged by symmetry or postoperative time alone.

This review has several limitations. Its narrative design, English-language restriction, and absence of a formal risk-of-bias assessment may introduce selection and interpretation bias. Differences in graft types, patient populations, assessment methods, rehabilitation protocols, and follow-up periods also limit comparisons between studies. Furthermore, improvements in strength or symmetry should not automatically be interpreted as evidence of reduced reinjury risk.

Future research should establish standardized strength and activation assessments, clarify clinically meaningful recovery targets, and compare rehabilitation strategies across graft types and patient groups. Longer follow-up is needed to determine whether improvements in quadriceps strength, rapid force production, and movement quality translate into sustained sporting participation and better long-term knee function.

## Conclusions

Persistent quadriceps weakness after anterior cruciate ligament reconstruction (ACLR) is a multifactorial impairment involving muscular, neural, surgical, and rehabilitation-related factors. Effective management requires individualized, criterion-based rehabilitation, integrating progressive strengthening with objective assessments of muscle strength, functional performance, and movement quality. Adjunctive interventions, such as neuromuscular electrical stimulation (NMES) and blood-flow restriction (BFR) training, may be considered according to individual needs. Ultimately, return-to-sport (RTS) decisions should be guided by comprehensive functional recovery and psychological readiness rather than time since surgery or limb symmetry alone.
